# Seroprevalence of human immunodeficiency virus in Nepalese blood donors: A study from three regional blood transfusion services

**DOI:** 10.4103/0973-6247.42663

**Published:** 2008-07

**Authors:** Bishnu Raj Tiwari, Prakash Ghimire, Surendra Karki, Manita Rajkarnikar

**Affiliations:** *Nepal Red Cross Society, Central Blood Transfusion Service, Kathmandu, Nepal*; 1 *Central Department of Microbiology, Tribhuvan University, Kathmandu, Nepal*

**Keywords:** Human immunodeficiency virus, Nepal, regional blood transfusion services, seroprevalence

## Abstract

**Background and Objective:**

The likelihood of human immunodeficiency virus (HIV) infection occurring in recipients of HIV seropositive blood is close to 100%. Transmission during window period is still possible even each unit of blood is tested for anti-HIV 1 and 2 antibodies. The possibility of window period transmission would be minimized if blood is collected from low risk targeted general public. A continuous surveillance data might prove valuable for concerned authorities to assess their service and plan for further improvements in transfusion safety. Our aim was to determine the seroprevalence of HIV in regional blood transfusion services located at three developmental regions of Nepal and compare the results.

**Materials and Methods:**

A total of 16,557 blood donors were screened for anti-HIV 1 and 2 antibodies in three blood transfusion services viz. 5,351 donors in Morang, 5,211 in Banke, 5,995 in Kaski by using rapid anti HIV 1 and 2 Test. The statistical significance of difference in seroprevalence was tested by Fisher’s Exact Test using the statistical software ‘Winpepi ver 3.8’.

**Results:**

The overall seroprevalence of HIV among blood donors in the regional blood transfusion services was 0.054% (9/16557) and 100% seropositivity was among male donors. The individual seroprevalence in Morang was 0.019%, in Banke was 0.095% and in Kaski was 0.05%. The HIV seroprevalence was not significantly different in regional blood transfusion services of Nepal (Fisher Exact Test, P = 0.2096).

**Conclusion:**

The seroprevalence in the regional blood transfusion service of Nepal was quite low and the seroprevalence rate was not significantly different.

## Introduction

The first case of acquired immune deficiency syndrome (AIDS) in Nepal was reported in 1988.[1] As of 15^th^ December 2007 National Center for AIDS and STD Control (NCASC) has officially confirmed 10,546 HIV positive cases in Nepal which include 1,610 confirmed cases of AIDS. It has also reported that among the 10,546 confirmed cases of HIV, 24 cases (0.23 %) are associated with blood transfusion or organ/tissue reception.[2] UNAIDS has estimated the adult (15-49 years) HIV prevalence rate of 0.5% by the end of 2005 in general population of Nepal, whereas the number of people living with HIV in the same time has been estimated to be 74,000.[3]

Globally, a total of 33.2 million (30.6-36.1 million) people were living with HIV in 2007, 6.3 million less than in 2006.[4] A total of 39.5 million (34.1-47.1 million) people were living with HIV in 2006, 2.6 million more than in 2004. In 2006 it has been reported that, in many part of the world, new HIV infections are heavily concentrated among young people.[5] Over the last few years HIV/AIDS epidemic in Nepal has gained ground and Nepal has progressed from a low prevalence country to one with so called concentrated epidemic in certain subgroups of the population. A situation analysis study of HIV/AIDS conducted in 2000 has identified the young people, Mobile populations, Female Sex Workers, Men who have sex with men, Injecting drug users, and Children as the most vulnerable to HIV/AIDS in Nepal.[6]

The likelihood of HIV infection occurring in recipients of HIV positive blood is close to 100% and the prevalence of HIV among recipients of blood or blood products before the screening of HIV in blood units was quite high. In countries where screening of blood for HIV has been instituted, the risk of HIV transmission through screened blood has been estimated to be 1/36000-1/225000 units transfused. This residual risk is reported to be due to antibody negative infected donors in the window period.[7] Present study was aimed to reveal the seroprevalence rate and compare the seroprevalence of HIV among blood donors from the three regional blood transfusion services situated in three developmental regions of Nepal.

## Materials and Methods

This was a descriptive cross-sectional study conducted in three regional blood transfusion services of Nepal over a period of one year from July 2006 - June 2007. Donors were selected if they fulfilled all the criteria to be eligible for donation as described by the Nepal Red Cross Society. All blood donors donating blood in respective blood transfusion services or in mobile camps were included in the study. Before drawing the blood, each donor was requested to fill a blood donors form. A total of 16,557 blood donors viz. 5,351 donors in Morang, 5,211 in Banke, 5,995 in Kaski were subjected for routine mandatory screening for Anti HIV 1 and 2 antibodies by an EIA based rapid test according the standard protocol described by respective company (HIV TRI-DOT, J. Mitra and Co, New Delhi, India). Initially reactive sera were reconfirmed by repeat testing. Blood samples were tested anonymously and confidentiality was maintained as described by Nepal Red Cross Society, Blood Transfusion Service. The significance of difference in seroprevalence of HIV among the three regional blood transfusion services was tested by ‘Fisher’s exact test’ using the software “Winpepi ver 3.8”

## Results

**Figure 1:**
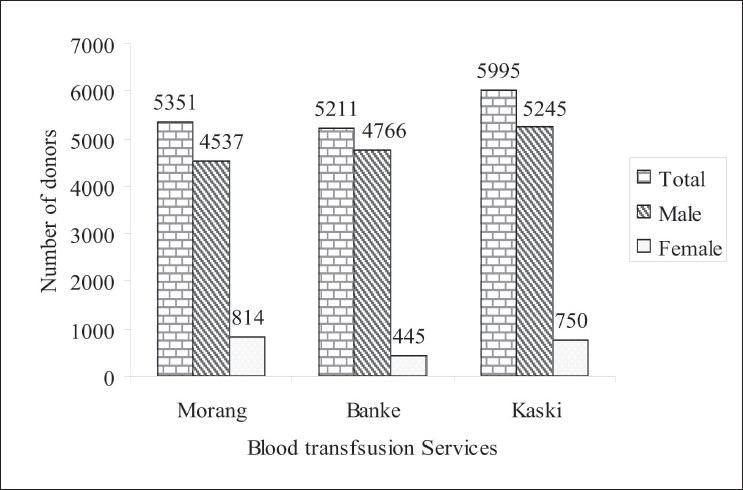
Demographic profile of study population

In the Morang (Biratnagar) Blood Transfusion Service, a total of 5,351 donors were screened in which 84.8% (4,537/5,351) were males and 15.2% (814/5,351) were females [Figure 1]. Among them only a single male donor was found seropositive giving the seroprevalence of 0.019% (1/5,351) [Table 1]. In the Banke (Nepalgunj) Blood Transfusion service, a total of 5,211 donors were screened in which 91.5% (4,766/5,211) were males and 8.5% were females [Figure 1]. Among them five donors were found seropositive giving the seroprevalence of 0.095% (5/5,211). All the seropositive donors were males with age ranging from 24-39 years [Table 1].

**Table 1:** Human immunodeficiency virus seropositivity status at three regional blood transfusion services of Nepal

Regional blood transfusion service	HIV seronegative	HIV seronegative	Fisher's exact test, *P*-value
Morang	5,350	1 (0.019%)	*P*= 0.2096
Banke	5,206	5 (0.095%)	
Kaski	5,992	3 (0.05%)	

HIV: Human immunodeficiency virus

In the Kaski (Pokhara) Blood Transfusion Service, a total of 5,995 blood donors were screened in which 87.5% (5,245/5,995) were males and 12.5% (750/5,995) were females [Figure 1]. Among them three donors were found seropositive giving the seroprevalence of 0.05% (3/5,995). All the seropositive donors were males with age ranging from 25-33 years [Table 1].

The difference in seroprevalence observed among the three regional blood transfusion services was not statistically significant (*P* = 0.2096, Fisher’s exact test).

## Discussion

This study revealed a low seroprevalence of HIV among the three regional blood transfusion services of Nepal compared with the seropositivity rate described for Kathmandu Valley (0.16%).[8] This difference might be due to the use of different test kits for screening, centralized urban setting of Kathmandu Valley or a reflection of actual epidemic in their targeted blood donor population. Similarly, Chander *et al*, has reported a 3.2% seroprevalence of HIV in patients attending teaching hospital from Bhairahava, Western Nepal which is much higher than the result of present study.[9 ] Particular vulnerability to HIV seropositivity was observed among male donors (100%) than among female donors which was also in accordance with the data of Chander *et al*. This might be due to the lower number of female donors in both of the studies. Our study revealed a quite low seroprevalence than described for general Nepalese population (0.5%)[3] and healthy Nepalese males (1.6%).[10] Such a significant difference might be due to stringent donor selection procedures implemented by Blood Transfusion Services. Similar lower seroprevalence among blood donors has been reported by Gupta *et al*,[11] from Ludhiana of India (0.084%), Ayala Gaylan *et al*,[12] from Mexico (0.02%), Rahaman *et al*,[13] from Pakistan (0.001%), Kakepoto *et al*,[14] from Pakistan (0.007%). This study revealed that the seroprevalence was not significantly different from one blood transfusion service to another despite the wide difference in geography and other factors associated with geographic variation. This study suggests that the policy applied by Nepal Red Cross Society, Blood Transfusion Service for recruiting and collecting the Safe blood donors is working equally well for all the regional blood transfusion services in Nepal.

## References

[1] Gurubacharya VL, Rana T, Subedi BK (2004). Profile of AIDS cases in Nepal. J Nep Med Assoc.

[2] National Centre for AIDS and STD Control (NCASC) (2007). Cumulative HIV and AIDS situation of Nepal. Final report.

[3] Unite Nation Development Program (UNDP) You and Aids: The HIV portal for South Asia-Nepal at a glance. http://www.youandaids.org/Asia%20Pacific%20at%20a%20Glance/Nepal/.

[4] United Nations Acquired Immuno Deficiency Syndrome (UNAIDS) AIDS epidemic. http://www.unaids.org/wad/2007/Epiupdate/.

[5] UNAIDS AIDS epidemic update. http://www.unaids.org/wad/2006/Epiupdate2006_.

[6] Pokharel BR, Aryal S, Bhattarai (2000). Situation analysis of HIV/AIDS in Nepal, National center for AIDS and STD control. Teku, Kathmandu: December, Final draft, Report.

[7] Folks TM, Khabbaz RF, Coller L, Balows A, Sussman M (1998). Retroviruses and associated diseases in humans. Topley and Wilson's microbiology and microbial infections. Virology.

[8] Nepal Red Cross Society Blood Transfusion Service. Annual Progress Report 2006/07.

[9] Chander A, Pawaha VK (2004). Seroprevalence of HIV-1/HIV-2 infection in Bhairahawa, Western Nepal: A hospital based study. J NHRC.

[10] Joshi SK, Ghimire GR (2003). Serological prevalence of Antibodies to Human Immunodeficiency Virus (HIV) and Hepatitis B Virus (HBV) among Healthy Nepalese Males: A retrospective study. Kathmandu Univ Med J.

[11] Gupta N, Kumar V, Kaur A (2004). Seroprevalence of HIV, HBV, HCV and syphilis in voluntary blood donors. Indian J Med Sci.

[12] Ayala Gaytán JJ, Guerra Avalos FJ, Mora Brondo P, Casillas Romo A (1997). Prevalence of viral markers for hepatitis B, C and human immunodeficiency virus in volunteer blood donors in northeast mexico. Rev Gastroenterol Mex.

[13] Rahman M, Akhtar G, Lodhi Y (2002). Seroprevalence of Hepatitis C antibodies in blood donors. Pak J Med Sci.

[14] Kakepoto GN, Bhally HS, Khaliq G, Kayani N, Burney IA, Siddiqui T (1996). Epidemiology of blood borne viruses: A study of healthy blood donors in Southern Pakistan. Southeast Asian J Trop Med Public Health.

